# ﻿An integrative approach to species delimitation sinks three Chinese limestone karst *Elatostema* (Urticaceae) species

**DOI:** 10.3897/phytokeys.236.114837

**Published:** 2023-12-06

**Authors:** Zi-Bing Xin, Alexandre K. Monro, Ren-Fen Wang, Long-Fei Fu

**Affiliations:** 1 Guangxi Key Laboratory of Plant Conservation and Restoration Ecology in Karst Terrain, Guangxi Institute of Botany, Guangxi Zhuang Autonomous Region and Chinese Academy of Sciences, Guilin 541006, China Guangxi Institute of Botany, Guangxi Zhuang Autonomous Region and Chinese Academy of Sciences Guilin China; 2 Identification & Naming Department, Royal Botanic Gardens, Kew TW9 3AE, UK Royal Botanic Gardens London United Kingdom

**Keywords:** DNA barcoding, integrative taxonomy, phylogenetically informative morphological characters, phylogeny, point-endemics

## Abstract

*Elatostema* is recognized as a taxonomically difficult group due to the reduced nature of the tiny flowers and inflorescences, also the large number of species (ca 650 to 700). Different opinions on morphological species delimitation have resulted in instability, which is problematic in such a speciose group. In this paper, the taxonomic status of three putative species, *E.robustipes*, *E.scaposum*, *E.conduplicatum* and their hypothetical closest relatives, was revised using morphological and molecular observations. Morphological comparison suggested high similarity between *E.robustipes* & *E.retrohirtum*, *E.scaposum* & *E.oblongifolium*, *E.conduplicatum* & *E.coriaceifolium*, respectively. Phylogenetic analyses of four universal DNA barcodes (ITS, *trnH*-*psbA*, *matK* and *rbcL*) suggested that each species pair represents a single evolutionary lineage. Taking these two findings together, we propose *E.robustipes* to be a synonym of *E.retrohirtum*, *E.scaposum* a synonym of *E.oblongifolium*, and *E.conduplicatum* a synonym of *E.coriaceifolium*. Our results recover the number, shape and size of the bracts and bracteoles to be relatively stable characters, and the disposition of the male inflorescences on modified stems to be an unstable character, unsuitable for species delimitation in *Elatostema*.

## ﻿Introduction

*Elatostema* J.R.Forst. & G.Forst. is one of the most species-rich genera in the Urticaceae, comprising ca 650 to 700 species of mainly succulent herbs that grow in dense shade of forest, stream sides, gorges, and caves (Wang 2014; POWO 2023). *Elatostema* is distributed throughout tropical and subtropical Africa, Madagascar, Asia, Australia and Oceania (Lin et al. 2003). Recent phylogenetic studies suggest that *Elatostema* is a monophyletic group that includes taxa hitherto attributed to *Pellionia*, but excludes those attributed to *Elatostematoides*, *Procris*, and *Pellioniarepens* (Tseng et al. 2019).

Southwest China and Southeast Asia are renowned biodiversity hotspots, in part due to extensive limestone karst present in both (Xu 1995; Clements et al. 2006; Wei 2018; Wei et al. 2022). Southwest China is the center of both *Elatostema* species diversity and morphological variation within the genus, which suggests that it may be a center of diversification for the genus (Wang 2014). This diversity is associated with limestone karst, a fragile habitat characterized by substantial exposed rock, shallow soils deficient in N and P, excessive Ca, Mg, seasonal droughts (Hao et al. 2015), high species diversity and point-endemism (Clements et al. 2006). Karst has been subject to extensive human intervention (mining, agriculture) and is very sensitive to climate change and water pollution (Parise et al. 2009; Jiang et al. 2014). Documenting this biodiversity is a high priority if species are to be conserved or their extinction threat assessed (Clements et al. 2006; Wang 2014). Collecting in karst is, however, very difficult as there are few roads and the terrain is irregular and steeply dissected. A consequence of the above is that there are relatively few collections from such areas and undescribed species are frequently represented by one or two collections only (Fu et al. 2019). Describing species based on few collections is problematic and carries the risk of over-describing species (Fu et al. 2019). Furthermore, considering the information lacking on pistillate inflorescence (21%) and staminate inflorescence (29%) of Chinese *Elatostema* species (Wang 2014), as well as the extensive occurrence of apomixis in *Elatostema* (Fu et al. 2017), it is likely that many new species are described from populations that comprise a single sex, further increasing the risk of over-description, whereby male and female populations can be described as different species.

Over the past two decades, there has been some instability in *Elatostema* species delimitation in China with several *Elatostema* species being placed in synonymy or reduced to ranks below that of species (Duan and Lin 2003; Lin and Duan 2003; Duan et al. 2006; Lin et al. 2011) and then raised back to species rank (Wang 2010a, 2014) because of different opinions on the importance of specific morphological characters. Wang (2010a) studied bract and bracteole morphology of *Elatostema* and concluded that their number, shape and size are relatively constant. More recently, additional morphological characters, such as male inflorescences borne on modified stems, were identified and used to delimitate species (Yang et al. 2011; Wang 2014). The phylogenetic informativeness of these characters has not, however, been evaluated (Lin et al. 2011). Controversy in species delimitation cannot, therefore, be resolved by morphological evidence alone.

Phylogenetic analyses of DNA barcodes can provide a means to use paraphyly to identify conspecific groupings and it has been successfully applied to delimit *Elatostemasetulosum* W.T.Wang (Fu et al. 2019).

In this study we evaluate the taxonomic status of three putative *Elatostema* species, *E.robustipes* W.T.Wang, F.Wen & Y.G.Wei, *E.scaposum* Q.Lin & L.D.Duan, *E.conduplicatum* W.T.Wang. To do so, we employed four universal DNA barcodes (ITS, *trnH*-*psbA*, *matK* and *rbcL*) (Hollingsworth et al. 2009, 2011; China Plant BOL Group et al. 2011) and visual morphological comparison. We aimed to use a phylogenetic framework to evaluate the phylogenetic informativeness of several morphological characters (see Wells et al. 2021) that have previously been used for species delimitation in the above species, as well as assess their monophyly.

## ﻿Materials and methods

### ﻿Taxon sampling

Fieldtrips in SW China and northern Vietnam were conducted between 2009 and 2023 to collect specimens and DNA materials of *Elatostemarobustipes*, *E.scaposum*, *E.conduplicatum* and their closest relatives for morphological and molecular studies. Our sampling strategy of molecular analysis aimed to sample all putative species and their closest relatives, using material collected from type localities or, where this was not possible, to sample material that exhibited the diagnostic morphological characters for those species. *Elatostemarobustipes* and *E.conduplicatum* were collected from type localities and *E.scaposum* from a specimen with key characters. The studied material covered the main distribution range of the respective species in SW China to northern Vietnam. In total, seven species with 11 accessions represented by three putative species and their closest relatives with the exception of *E.shanglinense* W.T.Wang (DNA material is not available) were ingroups. *Elatostemaradicans* (Siebold & Zucc.) Wedd. and *E.heterolobum* (Wedd.) Hallier f. were selected as outgroups based on previous phylogenetic analyses (Tseng et al. 2019). Materials for molecular analysis are listed in Table 1. The morphological comparison between putative species and their closest relatives were based on consulting protologues, checking type specimens and other specimens as well as observing field individuals. Specimens used for morphological studies are listed in Appendix 1.

**Table 1.** Species name, voucher specimen of *Elatostema* and their accession numbers of ITS, *trnH*-*psbA*, *matK* and *rbcL* used in this study (*denoted newly generated sequences).

Species name	Accession	Voucher specimen	Locality	ITS	*trnH*-*psbA*	*matK*	*rbcL*
*Elatostemabalansae*	C098	L.F. Fu & S.L. Huang FL0001 (IBK)	Yunnan, Guangxi	OR733575 ^*^	OR730813 ^*^	OR730814 ^*^	OR730815 ^*^
*E.conduplicatum*	C038	L.F. Fu FLF042 (IBK)	Guangxi, China	OR577332 ^*^	OR568594 ^*^	OR568577 ^*^	OR591476 ^*^
*E.coriaceifolium*	C696	F. Wen 0097 (IBK)	Guizhou, China	OR577335 ^*^	OR568595 ^*^	OR568585 ^*^	OR591473 ^*^
	C140	F. Wen WF0070 (IBK)	Guizhou, China	OR577334 ^*^	OR568596 ^*^	OR568583 ^*^	OR591468 ^*^
	J078	Z.B. Xin XZB20180128-01 (IBK)	Guangxi, China	OR577333 ^*^	OR568597 ^*^	OR568584 ^*^	OR591475 ^*^
*E.heterolobum*	C775	Y.G. Wei Wei054 (IBK)	Guangxi, China	OR577343 ^*^	OR568593 ^*^	OR568587	OR591474 ^*^
*E.oblongifolium*	C199	Y.G. Wei & F. Wen 1147 (IBK)	Guizhou, China	OR577338 ^*^	OR568588 ^*^	OR568582 ^*^	OR591469 ^*^
	C067	Y.G. Wei & L.F. Fu 068 (IBK)	Guangxi, China	OR577336 ^*^	OR568589 ^*^	OR568581 ^*^	OR591478 ^*^
*E.radicans*	C694	F. Wen 0111 (IBK)	Guizhou, China	OR577342 ^*^	OR568599 ^*^	OR568586 ^*^	OR591472 ^*^
*E.retrohirtum*	C598	A.K. Monro &Y.G. Wei AM6801 (IBK)	Guangxi, China	OR577340 ^*^	OR568591 ^*^	OR568579 ^*^	OR591470 ^*^
	C610	L.F. Fu & S.L. Huang FL0026 (IBK)	Yunnan, China	OR577341 ^*^	OR568592 ^*^	OR568580 ^*^	OR591471 ^*^
*E.robustipes*	C041	Y.G. Wei & L.F. Fu 002 (IBK)	Guangxi, China	OR577339 ^*^	OR568598 ^*^	OR568578 ^*^	OR591477 ^*^
*E.scaposum*	C085	F. Wen WF0068 (IBK)	Guizhou, China	OR577337 ^*^	OR568590 ^*^	/	OR591479 ^*^

### ﻿Morphological examination

A morphological species concept was employed to compare the taxa based on Wei et al. (2011). Specimens were examined using dissecting microscopy following Fu et al. (2019). The selection of morphological characters was made based on the morphological diagnosis of three putative species distinguishing from their closest relatives (Wang 2010b; Yang et al. 2011; Wei et al. 2012).

### ﻿Genomic DNA extraction, PCR amplification & sequencing

Four universal barcodes: the nuclear ribosomal internal transcribed spacer (ITS) region, the *trnH*-*psbA* intergenic spacer, *matK* and *rbcL* were used to establish hypotheses of phylogenetic relationship based on their ability to detect variation at the species level (China Plant BOL Group et al. 2011; Gao et al. 2012). The primers used to amplify four universal barcodes were those developed by the Kress et al. (2005), China Plant BOL Group et al. (2011) and Gao et al. (2012). Total genomic DNA was isolated from dried plant material using a modified CTAB protocol (Chen et al. 2014). PCR amplification protocols followed Gao et al. (2012) and Fu et al. (2019).

### ﻿Sequence alignment and phylogenetic analysis

Sequence data were edited and assembled using Lasergene Navigator (DNAStar, Madison, Wisconsin, USA). Cleaned sequences were then aligned with the MEGA 5.1 (Tamura et al. 2011) with additional manual refinements where necessary. Phylogenetic analyses for the aligned matrix were performed by maximum parsimony (MP), Bayesian inference (BI) and maximum likelihood (ML) methods. MP analyses were carried out using PAUP* 4.0b10 (Swofford 2002). All characters were unordered and equally weighted, and gaps were coded as missing data. Heuristic searches were performed using a starting tree built from stepwise addition with tree bisection-reconnection (TBR) branch swapping and 1,000 random addition replicates. To assess confidence in clades, 1,000 bootstrap replicates (maximum parsimony bootstrap; MPBS) with 10 random additions per replicate were used. The ML analyses were constructed in IQtree v1.6.12 (Nguyen et al. 2015) with 1,000 bootstrap replicates (MLBS) and HKY+G selected as the best model. The BI analyses were done using MrBayes v3.2.7a (Ronquist et al. 2012). The model of best fit (TIM+F+G4) was determined based on Bayesian information criterion (BIC) (Aho et al. 2014) in jModelTest2 v. 2.1.7 (Darriba et al. 2012). Two independent runs were performed, each consisting of four Markov Chain Monte Carlo (MCMC) chains. The beginning 25% of trees were discarded as burn-in while the remaining trees were used for generating a consensus tree to estimate posterior probabilities (PP). The convergence of the MCMC chains of each run was determined when the average standard deviation of split frequencies (ASDSF) achieved ≤ 0.01.

### ﻿Estimates of support

We adopted the same criteria of ML, MP and BI analyses as Tseng et al (2019): For ML and MP analyses, 70–79%, 80–89%, and 90–100% bootstrap supports were considered as weakly, moderately, and strongly supported, respectively, and values lower than 70% were considered as providing no support. For BI analyses, the posterior probabilities of < 0.9, 0.9–0.94, 0.95–0.99, and 1.0 were considered as providing no, weak, moderate, and strong support, respectively.

## ﻿Results

### ﻿Morphological comparison

After consulting protologues, checking type specimens and other specimens as well as observing field individuals, a suite of morphological characters was confirmed and used to compare three putative species and their closest relatives. Characters used were plant height, stem indumentum, leaf shape, leaf indumentum, leaf venation, stipule shape, male inflorescence insertion, male inflorescence peduncle length, male bract appendage, male bracteole shape and male flower sepal number. Specifically, *Elatostemarobustipes* had densely hispid stem comprising weakly curved to crooked, appressed hairs, broader half auriculate leaf basal, 3.5–4.5 mm male inflorescence peduncle length, 5 longitudinal ribs outer bracts and 1 longitudinal rib inner bracts, and oblanceolate or obovate male bracteoles that can be easily distinguished from *E.balansae* Gagnep., but showed no significant difference to *E.retrohirtum* Dunn (Table 2). *Elatostemascaposum* presented 50–90 cm plant height, male inflorescence borne on modified or unmodified stems and 5–15 mm male inflorescence peduncle length showing no significant difference to *E.oblongifolium* Fu ex W.T.Wang (Table 3). *Elatostemaconduplicatum* had glabrous leaf blade, semitriplinerve leaf venation, triangular stipule, broadly triangular and corniculate male bract, linear-cymbiform and corniculate male bracteoles and 5-merous male flower which can be easily distinguished from *E.shanglinense*, but showed no significant difference to *E.coriaceifolium* W.T.Wang (Table 4).

**Table 2.** Morphological comparison of *Elatostemarobustipes*, *E.balansae* and *E.retrohirtum*.

Characters	*E.robustipes*	*E.balansae*	*E.retrohirtum*
Stem indumentum	densely hispid, weakly curved to crooked	glabrous or pubescent	densely hispid, weakly curved to crooked
Leaf shape	broader-half auriculate	broader-half broadly cuneate or rounded	broader-half auriculate
Male inflorescence peduncle length	3.5–4.5 mm	1.0–2.0 mm	4.0–6.0 mm
Male bract appendage	outer bracts bearing 5 longitudinal ribs; inner bracts bearing 1 longitudinal rib	outer bracts bearing 3 inconspicuous longitudinal ribs; inner bracts not bearing rib	outer bracts bearing 5–6 longitudinal ribs; inner bracts bearing 1–3 longitudinal ribs
Male bracteoles shape	oblanceolate or obovate	linear	Oblanceolate

**Table 3.** Morphological comparison of *Elatostemascaposum* and *E.oblongifolium*.

Characters	*E.scaposum*	*E.oblongifolium*
Plant height	50–90 cm	20–90 cm
Disposition of male inflorescence	borne on modified or unmodified stems	borne on unmodified stems
Male inflorescence peduncle length	5–15 mm	0.5–10 mm

**Table 4.** Morphological comparison of *Elatostemaconduplicatum*, *E.shanglinense* and *E.coriaceifolium*.

Characters	*E.conduplicatum*	*E.shanglinense*	*E.coriaceifolium*
Leaf indumentum	glabrous	shortly ciliated	Glabrous
Leaf venation	semitriplinerve	triplinerve	Semitriplinerve
Stipule shape	triangular	narrowly lanceolate	triangular or narrowly ovate
Male bract shape	broadly triangular, conduplicate	oblong-cymbiform, not conduplicate	broadly ovate, conduplicate
Male bracteoles shape	linear-cymbiform, corniculate	narrowly linear, not corniculate	linear-cymbiform, corniculate
Male flower merism	5	4	5

### ﻿Molecular analysis

**Figure 1.**
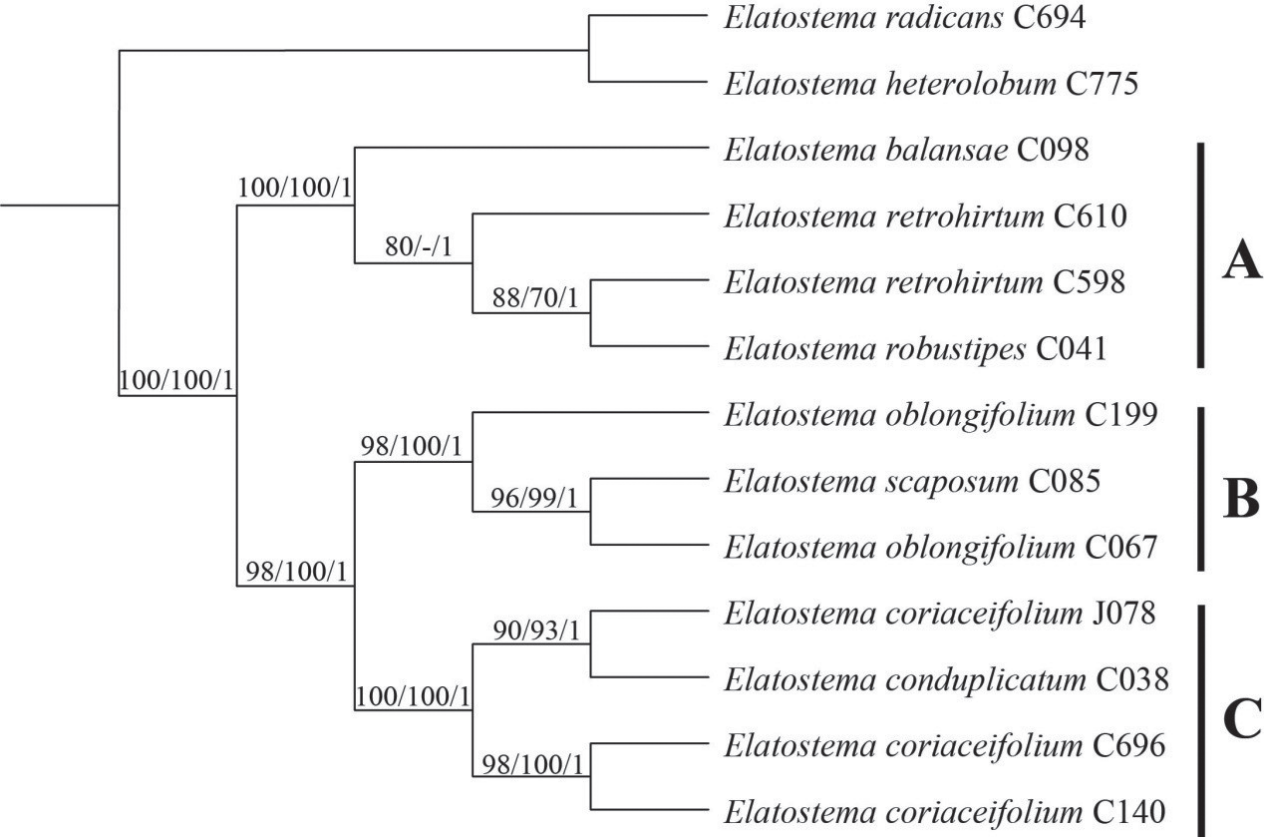
Phylogenetic tree of *Elatostema* generated by maximum likelihood (ML) of the combined dataset (ITS, *trnH*-*psbA*, *matK*, *rbcL*). Numbers on branches indicate bootstrap values (≥60%) of ML and maximum parsimony (MP) analyses and posterior probability (≥0.8) of Bayesian inference (BI).

The combined matrix had a length of 2,526 characters (ITS: 652, *trnH*-*psbA*: 375, *matK*: 800, *rbcL*: 699). Including indels, 289 (11.4%) were variable and 205 (8.1%) were parsimoniously informative. Phylogenies reconstructed using ML, MP and BI methods recovered consistent topologies for all samples of the ingroup taxa, which formed a monophyletic clade with strong supports (MLBS 100, MPBS 100, PP 1.0) sister to the outgroup taxa. The ingroup taxa were recovered as three subclades (A–C), each of which comprised the putative species and its most morphologically similar congener in a paraphyletic grouping (Fig. 1). In detail, clade A comprised three species including *Elatostemabalansae*, *E.retrohirtum* and *E.robustipes* with strong supports (MLBS 100, MPBS 100, PP 1.0). *Elatostemabalansae* was sister to a clade consist of *E.retrohirtum* and *E.robustipes*, whereas *E.robustipes* was nested in this clade including a paraphyletic *E.retrohirtum*, with strong supports in BI analysis (PP = 1). Similar situations also occurred in clade B and clade C that *E.scaposum* and *E.conduplicatum* were nested in the paraphyletic *E.oblongifolium* and *E.coriaceifolium*, respectively with strong supports (MLBS 98/100, MPBS 100/100, PP 1.0/1.0).

## ﻿Discussion

**Figure 2.**
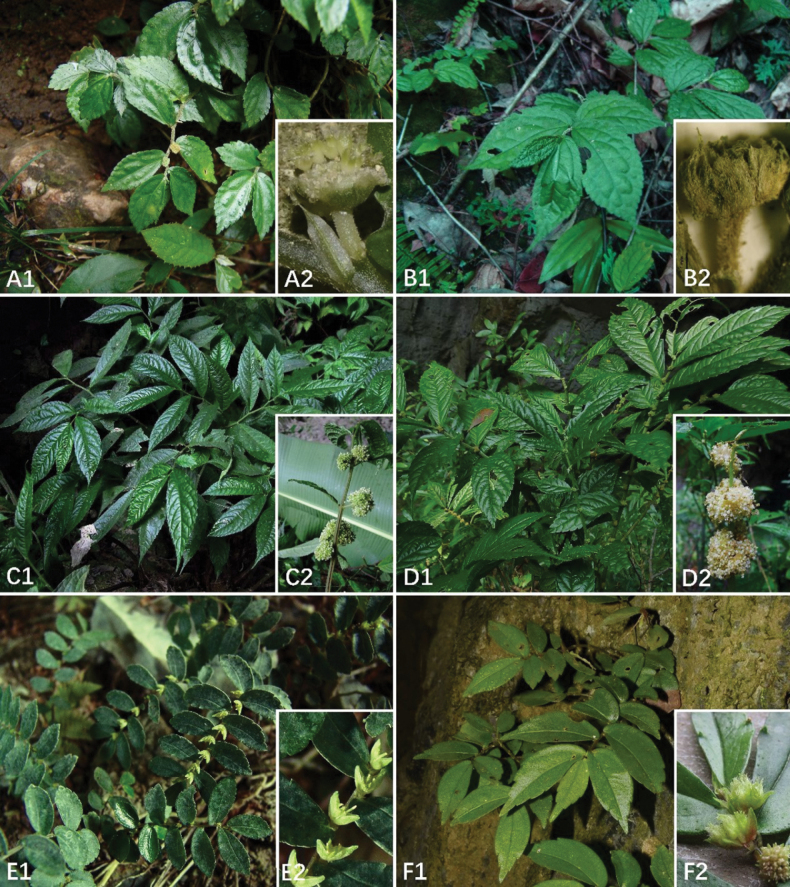
*Elatostema* spp. **A***E.retrohirtum***B***E.robustipes***C***E.oblongifolium***D***E.scaposum***E***E.coriaceifolium***F***E.conduplicatum***1** habit **2** male inflorescence.

*Elatostemarobustipes* was described based on a single collection with two duplicate specimens that displayed only male inflorescences, with reference to *E.balansae* (Wei et al. 2012). It was argued that *E.robustipes* could be distinguished from *E.balansae*, based on leaf shape, male inflorescence peduncle length, bract appendage and bracteole shape (Table 2). At the time of description, *E.robustipes* was not compared to another morphologically similar species, *E.retrohirtum*, as no description of the male inflorescence for the latter was known, limiting the basis of a comparison. During subsequent fieldtrips to Yunnan and northern Vietnam, we collected several specimens of *E.retrohirtum* bearing male inflorescences (Fu et al. 2014). These suggested that *E.robustipes* was, in fact, most similar to *E.retrohirtum*. A detailed morphological comparison of *E.robustipes* and *E.retrohirtum* showed that they share several diagnostic morphological characters: a densely hispid stem comprising weakly curved to crooked, appressed hairs; a male inflorescence with an involucre comprising six bracts, the outer two of which are larger and bear five longitudinal ribs, the four inner of which are smaller and bear a single longitudinal rib (Table 2; Fig. 2). Our phylogenetic analyses suggest that *E.robustipes* is nested in a clade A, which includes a paraphyletic *E.retrohirtum*, with strong supports in BI analysis (PP 1.0), and which itself is sister to a morphologically distinct, *E.balansae*. Based on the morphological and molecular evidence, we consider that *E.robustipes* and *E.retrohirtum* represent the same species. The latter name has priority.

*Elatostemascaposum* was described with reference to *E.oblongifolium*, based on 12 duplicate specimens (Yang et al. 2011). The diagnostic characters in the diagnosis were plant height, male inflorescence peduncle length and the disposition of the male inflorescences (Yang et al. 2011). Based on our extensive field observations and collections across Guizhou and Yunnan, plant height and male inflorescence peduncle length for both groupings overlapped, and a single population of *E.scaposum* being recovered which comprises individuals bearing male inflorescences on both modified and unmodified stems (Table 3; Fig. 2) suggesting that the character of modified stem is not stable for the species. Our phylogenetic analyses recovered *E.scaposum* nested in clade B, which includes a paraphyletic *E.oblongifolium*, with strong support (MLBS 98, MPBS 100, PP 1.0). Based on the morphological and molecular evidence, we consider that *E.scaposum* and *E.oblongifolium* represent the same species. The latter name has priority.

*Elatostemaconduplicatum* was described from two duplicate specimens of a single collection comprising only male inflorescences, with reference to *E.shanglinense* (Wang 2010b). *Elatostemaconduplicatum* can easily be distinguished from *E.shanglinense* by a suite of characters, including leaf indumentum and venation, stipule shape, male bract shape and conduplicate arrangement, male bracteole shape, and male flower merism (Table 4). *Elatostemaconduplicatum* was not, however, compared with another species, *E.coriaceifolium*, presumably as the male inflorescences of the latter were unknown at the time. Wang (2010c) later provided a supplementary description of the male inflorescences of *E.coriaceifolium*, which suggested that *E.conduplicatum* was most similar to *E.coriaceifolium*, from which it could be distinguished by the conduplicate bract arrangement. Our field observations, however, clearly showed that the male bract of *E.coriaceifolium* is also conduplicate (Table 4, Fig. 2). In addition, our phylogenetic analyses suggest that *E.conduplicatum* is nested within clade C, which includes a paraphyletic *E.coriaceifolium*, with strong supports (MLBS 100, MPBS 100, PP 1.0). Based on the morphological and molecular evidence, we consider that *E.conduplicatum* and *E.coriaceifolium* represent the same species. The latter name has priority.

Based on the above, we present the following detailed taxonomic treatments for the species.

### ﻿Taxonomic treatment

#### ﻿ Elatostema coriaceifolium

Taxon classificationPlantaeRosalesUrticaceae

W.T.Wang, Acta Phytotax. Sin. 31(2): 170. 1993.

02728CB7-5052-52FC-8B18-0967E0930514

= Elatostema conduplicatum W.T.Wang, Guihaia 30(1): 3. 2010. Syn. nov. Type: China. Guangxi: Donglan County, Bala, *Y.M. Shui & W.H. Chen B2004-171A* (holotype: PE [PE01842427!]; isotype: KUN!).

##### Type.

China. Guizhou: Libo, Wengangmogan, 29 April 1984, *Q.H. Chen et al. 2289* (holotype: HGAS).

##### Description.

***Perennial herb*.** Stems 140–185(–270) × 1.0–1.5 mm, erect, simple, fasciculate, furfuraceous, glabrous; stipules 2, triangular or narrowly ovate, 0.8–1.8 × 0.4–1.0 mm, without cystoliths. Leaves sessile or shortly petiolate, glabrous; laminae 12–24(–40) × 8–11(–14) mm, obliquely elliptic or rhombic-elliptic, chartaceous or thinly coriaceous, semitriplinerve; cystoliths densely scattered; base asymmetrical, broader-half auriculate, narrower-half cuneate; margin denticulate; apex acute. Staminate inflorescences solitary, capitate; sessile; receptacle inconspicuous, subtended by marginal bracts; the bracts 6, unequal, outer 2 bracts major, broadly ovate, conduplicate, 4–4.5 × ca. 3 mm, abaxial surface with 1 longitudinal rib, ribbed extending apically as a corniculate protuberance, inner 4 bracts minor, oval, ca. 3 × 2–2.8 mm, abaxial surface with 1 longitudinal rib, ribbed extending apically as a corniculate protuberance, glabrous; staminate flowers pedicellate; bracteoles 2 per flower, subequal, ca. 2.4 × 1.0 mm, linear-cymbiform, subapical appendage, corniculate, shortly ciliate; tepals 5, broadly ovate, subapical appendage 0.8–1.1 mm, corniculate, pubescent. Pistillate inflorescence solitary, capitate; peduncle ca. 1 mm, glabrous; receptacle broadly rectangular, 3–3.5 mm, glabrous, subtended by marginal bracts, the bracts ca. 20, unequal, outer 6 bracts major, triangular or broadly ovate, ca. 1.1 × 0.4–2 mm, subapical appendage, inner bracts minor, narrowly triangular or linear, sparsely ciliate or glabrous; pistillate flowers pedicellate; bracteoles 2 per flower, equal, 0.9–1.5 × 0.1–0.4 mm, linear, ciliate or glabrous; achenes ca. 0.8 mm, narrowly ellipsoidal, ca. 6–8-ribbed.

##### Distribution.

This species is endemic to China (Guangxi, Guizhou).

#### ﻿ Elatostema oblongifolium

Taxon classificationPlantaeRosalesUrticaceae

Fu ex W.T.Wang, Bull. Bot. Lab. N. E. Forest. Inst., Harbin 7: 26. 1980.

974A881E-77C6-54BE-9490-E5F6DEA61D00

≡ Pellionia bodinieri H. Lév., Repert. Spec. Nov. Regni Veg. 11: 551. 1913.
≡ Elatostema bodinieri (H.Lév.) Hand.-Mazz., Symb. Sin. Pt. 7: 144. 1929, nom. illeg., non H.Lév. 1913. Type: China. Guizhou: Gan-pin, 29 April 1897, *Bodinier 1547* (syntype E); Ou-la-gay, 9 April 1898, *Seguin s.n.* (syntype: E).
= Elatostema schizocephalum W.T.Wang, Bull. Bot. Lab. N. E. Forest. Inst., Harbin 7: 82. 1980. Type: China. Hunan: Yizhang, 22 January 1942, *S.Q. Chen 73* (holotype: PE [PE00023194!]). = Elatostemamulticanaliculatum B.L.Shih & Yuen P. Yang, Bot. Bull. Acad. Sin. 36: 268. 1995. Type: China. Taiwan: Taoyuan Co., Mt. Lala, 23 October 1994, *B.L. Shih 3226* (isotypes: HAST, TAI!, TAIF).
= Elatostema scaposum Q. Lin & L.D. Duan, Nordic J. Bot. 29: 420. 2011. Syn. nov. Type: China. Guizhou: Libo County, Jialiang Baibidong, alt. 800 m, 26 October 2003, *Q. Lin & L.D. Duan 1023* (holotype: PE [PE01863021!]; isotypes E!, GH, HUH [HUH A00293663!], K, L, NY, PE [PE01863023!, PE01863023!], TUS, US, WU).

##### Description.

***Perennial herb*.** Stems 20–90 × 0.5–12 mm, ascending or erect, branched or simple, with 5 or more longitudinal canals, glabrous; stipules 2, narrowly triangular to subulate or narrowly lanceolate, 2.5–12 × 0.2–2.0 mm, glabrous. Leaves sessile or short petiolate; laminae 50–220 × 14–50(–80) mm, obliquely oblong or elliptic, chartaceous, pinnately nerved; cystoliths densely scattered; base asymmetrical, broader-half rounded to cordate, narrower-half cuneate; margin serrulate to coarsely serrate; apex acuminate or long acuminate. Staminate inflorescences borne on modified or unmodified stems, solitary, cymiferous, shortly pedunculate, subglabrous; bracts membranous, ovate, lanceolate or linear, 2–12 mm, glabrous; staminate flowers pedicellate, glabrous; tepals 5, narrowly elliptic, ca. 2 mm, subapical appendage, shortly corniculate. Pistillate inflorescence paired, capitate; peduncle ca. 3 mm, glabrous; receptacle rectangle or broadly ovate, deeply divided into two lobes, lobe further weakly divided into two lobes, 2–10 × 3 mm, glabrous, subtended by marginal bracts, the bracts 25 or more, unequal, outer bracts major, triangular, 0.6–1.0 × 0.4–0.8 mm, glabrous, inner bracts minor, linear or lanceolate, sparsely ciliate; pistillate flowers pedicellate; bracteoles 2 per flower, subequal, 0.5–1.5 mm, linear, narrowly obovate or cymbiform; achenes 0.6–0.9 × 0.3–0.5 mm, broadly ellipsoidal or ovoid, ca. 6-ribbed.

##### Distribution.

This species is distributed in China (Chongqing, Hubei, Hunan, Guangxi, Guizhou, Sichuan, Taiwan, Yunnan) and Vietnam (Ha Giang).

#### ﻿ Elatostema retrohirtum

Taxon classificationPlantaeRosalesUrticaceae

Dunn, Bull. Misc. Inform. Kew, Addit. Ser. 10: 249. 1912.

FFBFFF06-E03B-5023-96D2-50AD412D2EB1

= Elatostema robustipes W.T.Wang, F.Wen & Y.G.Wei, Ann. Bot. Fenn. 49: 188. 2012. Syn. nov. Type: China. Guangxi: Huanjiang County, Mulun National Reserve, Hongdong, alt. 308–512 m, 24°43'N, 108°18'E, 26 April 2009, *Y.G. Wei 124* (holotype: IBK!; isotypes: IBK!, PE [PE01843378!, PE01843379!]).

##### Type.

China. Guangdong: near Yit-hai Han valley, *Dunn’s Han Exped., Herb. Hongk. no. 6288* (holotype: K!).

##### Description.

***Perennial herb*.** Stems 150–350 × 1.8–2.5 mm, ascending or erect, branched, densely hispid, the hairs weakly curved to crooked, appressed; stipules 2, linear-lanceolate, 4–8 × 1.0–2.0 mm, cystoliths sparsely scattered, glabrous. Leaves sessile or short petiolate, petioles 0–1(–4.5) mm, densely hispid, the hairs weakly curved to crooked, appressed; laminae 40–60(–100) × 15–20(–50) mm, obliquely elliptic, herbaceous or chartaceous, triplinerve; cystoliths densely scattered; base asymmetrical, broader-half rounded or auriculate, narrower-half cuneate; margin denticulate; apex short acuminate or acute, rarely acuminate. Staminate inflorescences solitary, capitate; peduncle 3.5–6.0 × 0.3–0.8 mm, sparsely hispid, the hairs weakly curved, appressed; receptacle 2–4 × 3–5 mm, rectangle or oblong, glabrous, subtended by marginal bracts; the bracts ca. 6, unequal, outer 2 bracts major, broadly ovate, 2–2.5 × 4–5 mm, abaxial surface sparsely hispid, the hairs weakly curved, appressed, with 5 or 6 longitudinal ribs, each ribbed extending apically as a corniculate protuberance, inner 4 bracts minor, obovate, ca. 2 × 3 mm, abaxial surface with 1–3 longitudinal ribs, at least one ribbed extending apically as a corniculate protuberance, glabrous; staminate flowers pedicellate, glabrous; bracteoles 2 per flower, equal, 2.5–4.0 × 1.0–1.5 mm, oblanceolate or obovate, glabrous; tepals 4, ovate, 0.9–1.2 × 0.7–0.9 mm, subapical appendage ca. 0.5 mm, corniculate, glabrous. Pistillate inflorescence solitary, capitate; peduncle ca. 1 × 0.5 mm, glabrous; receptacle subrounded, 3–3.5 mm in diam., glabrous, subtended by marginal bracts, the bracts numerous, subequal, triangular, 0.6–1.2 × 0.4–0.6 mm; pistillate flowers pedicellate; bracteoles 2 per flower, subequal, ca. 0.6–1.2 × 0.3 mm, spatulate-linear; achenes 0.5–0.6 mm, ovoid or ellipsoidal, ca. 6-ribbed.

##### Distribution.

This species is distributed in China (Guangdong, Guangxi, Guizhou, Sichuan, Yunnan) and Vietnam (Bac Kan, Gia Lai, Ha Giang, Ha Noi, Hai Phong, Hoa Binh, Lam Dong, Nghe An, Ninh Binh, Son La, Tuyen Quang).

## ﻿Conclusions

We conclude that the three species studied are conspecific to earlier described taxa that have priority under the International Code of Nomenclature for Algae, Fungi and Plants and place them in synonymy with these names. Our results emphasize that some morphological characters, such as the number, shape and size of bract and bracteole, are relatively constant (Wang 2010a), whilst the bearing of male inflorescences on modified stems is unstable and ill-suited to delimit species in *Elatostema*. More importantly, our results provide further support for the need to integrate multiple lines of evidence when describing new species based on very small numbers of individuals, as is frequently the case for point-endemic species (Hong 2016; Fu et al. 2021).

## Supplementary Material

XML Treatment for Elatostema coriaceifolium

XML Treatment for Elatostema oblongifolium

XML Treatment for Elatostema retrohirtum

## ﻿Additional information

### Author ORCIDs

Zi-Bing Xin https://orcid.org/0000-0002-0062-6930

Long-Fei Fu https://orcid.org/0000-0001-8708-4718

### Data availability

All of the data that support the findings of this study are available in the main text.

## Appendix

Specimens used for morphological studies.

***Elatostemaconduplicatum*** W.T.Wang, **CHINA. Guangxi**: Donglan County, Bala, *Y.M. Shui & W.H. Chen B2004-171A* (KUN, PE [PE01842427]).

***Elatostemacoriaceifolium*** W.T.Wang, **CHINA. Guangxi**: Fengshan County, Yangzi cave, 28 January 2018, *Z.B. Xin XZB20180128-01* (IBK); **Guizhou**: Libo County, Jiarong Town, 21 October 2012, *F. Wen 0097* (IBK); Dushan County, Jichang Town, 19 October 2012, *F. Wen 0070* (IBK).

***Elatostemaoblongifolium*** Fu ex W.T.Wang, **CHINA. Guizhou**: Anlong County, 29 October 2010, *F. Wen 1082* (IBK); Dushan County, Jichang Town, 19 October 2012, *F. Wen 0068* (IBK); **Yunnan**: Guangnan County, Sanla waterfall, 08 November 2011, *F. Wen WFLTC111108* (IBK); Malipo County, Xiajinchang village, 23 October 2023, *C. Xiong & X.Y. He XC20231023-06* (IBK).

***Elatostemaretrohirtum*** Dunn, **CHINA. Guangdong**: near Yit-hai Han valley, *Dunn’s Han Exped., Herb. Hongk. no. 6288* (K); **Guangxi**: Longzhou County, 20 May 2010, *A.K. Monro & Y.G. Wei 6801* (IBK); **Yunnan**: between Malipo County and Babu Village, alt. 931 m, 23°14'52"N, 104°46'37"E, 2 May 2013, *L.F. Fu & S.L. Huang FL0015* (IBK).

***Elatostemarobustipes*** W.T.Wang, F.Wen & Y.G.Wei, **CHINA. Guangxi**: Huanjiang County, Mulun National Reserve, Hongdong, alt. 308–512 m, 24°43'N, 108°18'E, 26 April 2009, *Y.G. Wei 124* (IBK, PE[PE01843378, PE01843379]).

***Elatostemascaposum*** Q.Lin & L.D.Duan, **CHINA. Guizhou**: Libo County, Jialiang Baibidong, alt. 800 m, 26 October 2003, *Q. Lin & L.D. Duan 1023* (E, HUH [HUH A00293663], PE [PE01863021, PE01863023]).
